# Normalization of endometrial histopathology and endometrial NK cells concentration predict successful pregnancy in repeated implantation failure

**DOI:** 10.5935/1518-0557.20200049

**Published:** 2021

**Authors:** Alberto E. Tersoglio, Dante R. Salatino, Sebastian Tersoglio, Matías Castro, Adriana Gonzalez

**Affiliations:** 1 Private setting, International Center for Assisted Reproduction, Mendoza, Argentina; 2 Immunology Laboratory, Academics Units, National University of Cuyo, Mendoza, Argentina

**Keywords:** RIF, chronic endometritis, endometrial histopathology, endometrial NK, oocyte donation, endometrial flow cytometry

## Abstract

**Objective:**

The primary objective was to establish the endometrial predictors of clinical pregnancy in a population of repeated implantation failure with oocyte donation after specific endometrial treatment. The secondary one was to evaluate reproduction outcomes in terms of Implantation rate (IR), Clinical pregnancy (CP), Live birth delivery rate (LBDR) and Prematurity, in relation to normalization or no-normalization of the predictors.

**Methods:**

66 patients were assigned to the study. We ran a Pipelle endometrial biopsy to investigate the endometrium lymphocyte population by Flow Cytometry and abnormal/normal patterns by histopathology in pre/post-treatment. We employed the binary logistic regression model to identify the predictors for CP. For the secondary objective, we assessed the clinical outcomes in function to the normalization or no normalization in post-treatment.

**Results:**

Endometrial histopathology and endometrial NK cell counts resulted in CP predictors (Wald chi^2^ test (*p*=0.044 and 0.001)), respectively. We had a higher IR, CP and LBDR when both predictors were normalized in comparison with no normalization (*p*<0.001). There was a high percentage of prematurity in both normalized *vs.* non-normalized groups (34.4% (11/32) and 71.43% (5/7), respectively) without significant differences.

**Conclusion:**

Endometrial histopathology and endometrial NK cell counts showed that they are valid predictors of pregnancy outcome in repeated implantation failure after treatment. In post-treatment, the pregnancy outcomes were significantly higher in the presence of both normalized predictors. Pregnancy rates were zero in the no-normalization of both predictors. There was a high percentage of prematurity in both groups.

## INTRODUCTION

Repeated Implantation Failure (RIF) is a new nosological entity in the field of Assisted Reproduction, and it is the subject of study by both clinicians and researchers. Once the endometrium is isolated as the main subject, it is challenging to investigate what tools the clinician has to define in order to optimize the endometrium, and if they are efficient in terms of clinical pregnancy and live births in an RIF population in oocyte donation (OD). There is no universal definition of RIF, but according to published criteria, it could be defined as an absence of implantation after (3 consecutive cycles in IVF/ICSI or cryotransfer (embryo frozen replacement), where the cumulative number of embryos transferred was not less than three blastocysts, all of excellent quality in women <35 years of age and four or more good quality embryos in women (35 years, a concept that can be extended to egg donation ^(Polanski et al., 2014; Coughlan et al., 2014)^. One in every nine couples in Europe and the USA is affected by implantation disorders, and it is estimated that RIF occurs in 15-20% of in vitro fertilization-embryo transfers ^(Teklenburg et al., 2010; Cicinelli et al., 2008)^. Dismissing embryo quality as a critical factor in the implantation success, the endometrium becomes the principal object of study ^(Strowitzki et al., 2006)^.

Difficulties in human research to evaluate endometrial peri-implantation without compromising the nesting lead us to find new models of clinical research to address implantation failures. Egg donation is an attractive model to investigate the endometrium in patients with RIF ^(Zenke & Chetkowski, 2004)^. The implantation niche results from the interaction of multiple cells and effects in active and controlled processes ^(Navot et al., 1991; Mor & Cardenas, 2010; Bryceson et al., 2006; Nagamatsu & Schust, 2010; Lachapelle et al., 1996; Quenby et al., 2009; Mjösberg et al., 2007; 2010)^. The endometrial NK cells (enNK), the main constituent of the lymphocytic population in the endometrial mucosa, have been extensively studied in women with RIF and in those with repeated fetal loss; concluding that the enNK cells deregulation could be relevant in these cases ^(Beer et al., 1996; Coulam & Roussev, 2003)^.

The high association between Chronic Endometritis (CE) and RIF is established (14-31%), as well as unknown etiologies (28%) and recurrent pregnancy loss (9-13%) ^(Johnston-MacAnanny et al., 2010; Cicinelli et al., 2005; Tersoglio et al., 2015; Bouet et al., 2016; Kitaya et al., 2017)^. Endometritis is an infectious and inflammatory disorder of the endometrium. Although there are currently no universally accepted standardized definitions or diagnostic guidelines for CE, the literature agrees that the presence of multiple endometrial stromal plasmatic cells (PCs) is the most specific and sensitive finding in this pathology ^(Akopians et al., 2015; Michels, 1995; Kitaya *et al*., 2016)^. Endometrial immunohistochemistry (enIHQ) by CD138, a cell surface proteoglycan that is expressed on plasma cells, improves diagnostic accuracy ^(Kitaya & Yasuo, 2013)^. The CD138, being the prototype member of the syndecan family, is able to bind to interstitial collagen, fibronectin, members of the fibroblast growth factor (FGF-2), but also shows a clear biphasic pattern in respect to the menstrual cycle ^(Inki, 1997)^. CD138 decreases the amount of time spent looking for plasma cells in suspicious cases of CE, thus being the most reliable diagnostic method ^(Bayer-Garner & Korourian, 2001)^. Some caution should be exercised in its use and interpretations of results, since the normal endometrial epithelium can express CD138, the thickness of endometrial sections, the stage of the cycle and method of endometrial sampling ^(Inki, 1997; Torlakovic et al., 2015)^.

The cycle stage impacts PC prevalence, the endometrial sample obtained in the proliferative phase shows 50% higher PCs than in the secretory phase ^(Song et al., 2018)^. A possible explanation for such difference is that PCs tend to reside in the deeper layer of the endometrium in the proliferative stage ^(Kitaya & Yasuo, 2011)^. There is no consensus on PCs diagnostic criteria to define CE. Different criteria have been proposed in the literature, including at least one PC per section ^(Cicinelli et al., 2005)^, one PC per high power field (HPF) ^(Johnston-MacAnanny et al., 2010)^, one PC per ten HPFs ^(Kitaya & Yasuo, 2010)^, five PCs per ten HPFs ^(Bouet et al., 2016)^, five PCs per 20 HPFs ^(Kitaya & Yasuo, 2011)^, the presence of one to five PCs per HPF or discrete clusters of <20 PCs ^(Achilles et al., 2005)^, and an endometrial stromal plasmocyte density index (ESPDI>0.25), which results from the sum of the stromal CD138 cell count divided by the number of HPFs evaluated ^(Kitaya *et al*., 2017)^. On the other hand, a recent publication concluded that the concentration of plasma cells measured per unit area in EC was not greater than what we see in the fertile population ^(Liu et al., 2018)^.

Today, no conventional endometrial histopathology parameters are considered in the CE diagnosis, such as: superficial mucosal edematous changes, elevated stromal cell density, spindled stroma, dissociated maturation between epithelial cells/stromal fibroblasts, and polymorphic inflammatory cells - commonly associated with delayed differentiation of endometrium in the mid-secretory phase; pseudostratification and mitotic nuclei in both glandular and surface epithelial cells; even in the absence of increased plasma cells ^(Johnston-MacAnanny et al., 2010; Michels, 1995; Kitaya & Yasuo, 2010; Greenwood & Moran, 1981; Eschenbach, 2008; Mishra et al., 2008; Kiviat et al., 1990)^. One of the histomorphological characteristics of CE is a delayed differentiation of the endometrium in the mid-secretory phase (out of phase) ^(Kitaya & Yasuo, 2011)^. It is likely that estrogen and progesterone receptors are significantly increased in the inflamed endometrium ^(Mishra *et al*., 2008)^. There is a strong association between endometrial micropolyposis and CE. The stromal edema and increased vascularity produce the ‘hillocks’ in the hysteroscopy ^(Cicinelli et al., 2005)^. In a previous study, there was a high association of endometrial micropolyposis in CE (42.8%), and only one case without CE (6%) ^(Tersoglio & Salatino, 2017)^. An exhaustive search for plasma cells may not be necessary in the absence of these secondary characteristic findings ^(Greenwood & Moran, 1981)^.

Although it is generally accepted that the microorganisms frequently detected in endometrium with CE are common bacteria, such us: *Streptococcus* species, *Escherichia coli, Enterococcus faecalis, Staphylococcus* species, Mycoplasma/ureaplasma, *Chlamydia trachomatis, Proteus* species, *Gonococcus, Klebsiella pneumoniae, Pseudomonas aeruginosa, Gardnerella vaginalis, Corynebacterium*, and yeasts (*Saccharomyces cerevisiae* and *Candida species*) ^(Cicinelli et al., 2008; Kitaya et al., 2017)^. The endometrium microbiota and its alterations currently appear as a new approach in the CE etiopathogenesis. Even in the lack of endometrial pathogenic microorganisms in CE and RIF, some authors have proposed the use of antibiotics and control biopsies ^(Johnston-MacAnanny et al., 2010; McQueen et al., 2015)^. The evaluation of the endometrium by ultrasound is an accessible and reproducible method in assisted reproduction. Endometrial thickness (Eth) <7 mm or >14 mm, is established as inadequate for embryo transfer ^(El-Toukhy et al., 2008)^.

### Objectives

The primary objective was to find the endometrial predictors of clinical pregnancy (CP) in a population of repeated implantation failure (RIF) in oocyte donation (OD), after specific endometrial treatment. The secondary goal was to evaluate the reproductive results in terms of implantation rate (IR), clinical pregnancy (CP), live birth delivery rate (LBDR) and prematurity, in relation to predictors normalization.

## MATERIALS AND METHODS

### RIF Definition

We consider RIF as the absence of implantation after three or more cycles of IVF/ICSI, or freezing embryo transfer, where the cumulative number of embryos transferred was no less than 3/4 blastocysts with optimal quality, according to the Istanbul consensus criteria ^(Alpha Scientists in Reproductive & ESHRE special interest group of Embryology, 2011)^.

### Patient selection and details

**Figure 1:**
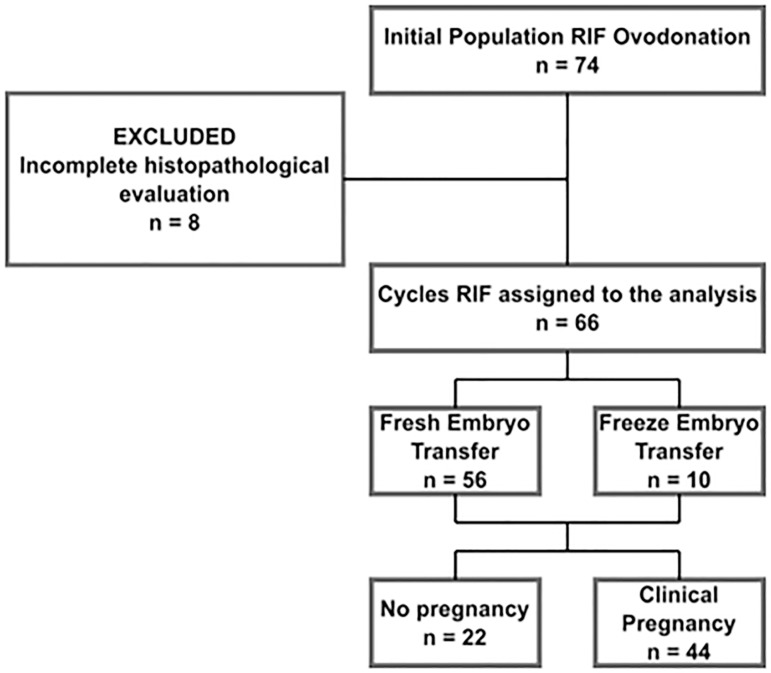
Flow diagram showing the case-definition method for 66 cases of RIF selected from a cohort, which used IVF/ICSI in egg donation in the period between 2008-2016

Over an initial population of n=74 cases of RIF in oocyte donation in the period 2008-2016, we analyzed 66 cycles/patient. The previous exclusion criteria were uterine malformation; autoimmune thyroid disease; thrombophilia; abnormal uterine cavity ascertained by hysterosalpingography, hysteroscopy or hysterosonography, and endometrial polyps. Of the 77 cases chosen, we excluded 7 because they did not complete endometrial histopathology (enHP) and endometrial flow cytometry (enFC) pre and post treatment. Of the total cycles/patient transferred n=66, 56 cycles were fresh embryo transfers, and 10 cycles were frozen-thawed embryo transfer (Figure 1).

Of the 66 cycles selected, the patients’ mean age was 39.46±4.91 (26-56) years, length of sterility 10(6 years, and a number of previous cycles ART (4±2.24). During the pretreatment, we measured endometrial thickness (Eth) on day 5 of the LH peak day in the spontaneous cycle, or on day 5 of exogenous progesterone use in the replacement cycle; resulting in an average of 10.52±2.08 mm, of which 23/66 (34.85%) showed ≤7 mm. Endometrial bacteriology was positive in 19/66 (28.79%), identifying mycoplasma hominis (8/19 (42.10%), chlamydial trachomatis 6/19 (31.58%) and Gram (-) & (+) bacteria 5/19 (26.32%). The pretreatment endometrium histopathology was normal in 13/66 (19.70%) and abnormal in 53/66 (80.3%). Of the abnormal enHP, 38/53 (71.7%) corresponded to CE, and the remaining 15/53 (28.3%) only showed abnormal enHP patterns. The length of therapy (days) calculated from the beginning of the treatment to the post-treatment evaluation showed a mean of 105 SD±63(42-168) days (Table 1).

**Table 1:** Patient and clinical characteristics in the RIF group before treatment

Patient and clinical characteristics	
Number of cycles	66
Number of previous IVF cycles w/failure	4.0±2.24 (2-11)
Patient age (year)	38.97±4.25 (29-52)
Length of sterility (year)	10.12±6.08 (1-20)
Endometrial thickness (mm)	10.52±2.08 (6-17)
Thinned endometrium (<7 mm)	23/66 (34.85%)
Positive endometrial bacteriology	19/66 (28.79%)
-*Mycoplasma genitalium*and*Ureaplasma urealyticum*	08/19 (42.10%)
-Chlamydia	06/19 (31.58%)
-Gram(-) & (+)^e^	05/19 (26.32%)
Endometrial histopathology	
Normal	13/66 (19.70%)
Abnormal	53/66 (80.30%)
-Chronic Endometritis	38/53 (71.7%)
-Histopathological Abnormal Pattern	15/53 (28.3%)
Length of therapy (days)	105±63 (42-168)

Note: Values were represented by median ± SD or (%) case/total.

e 1*Streptococcus*, 2 *Enterococcus Faecalis*, 1 *Eikinella cannis*, 1 *Enterococcus beta-hemolytic*.

### Endometrium biopsy, enHP, enFC, bacteriological and ultrasound parameters.

The evaluation by endometrium biopsy in pre and post-treatment was performed at day 5 of LH in the natural cycle or day 5 of exogenous natural progesterone use in a dose of 400 mgr/d vaginally, with previous variable doses of estradiol (6 to 10 mg/d) orally for 18 days in replacement cycle. We made the biopsies using the Cornier’s pipelle (Endomed^(^, Lab, Argentina) with axial movements of the entire endometrial surface in order to ensure an ideal intake. We used all the samples to investigate the lymphocyte population by CF and abnormal patterns by histopathology. In cases with favorable evolution, but the normalization was partial, we required a third biopsy. All patients gave their consent for performing endometrial biopsies, as well as polyvalent therapy in accordance with the regulations in force to date. We performed cytometric evaluation by CyFlow^®^ Counter two lasers, four colors and six parameters (Bexton Dickinson). We considered the following enFC variables; lymphocyte/cell population (Li/CP); T-lymphocytes, B-lymphocytes and NK cells over Li (LT/Li, LB/Li, NK/Li, respectively); CD3+CD4+ and CD3+CD8+ in relation to LT; CD4/CD8, and CD56/CD16 subpopulations. We established a normal reference group for the FC (n=25), corresponding to oocyte donors with a normal reproductive history (absence of abortions and normal live births), no history of vaginosis, negative endometrial bacteriology, normal enHP and enIHQ. We applied the same biopsy technique. We checked the data distribution of the reference group using the Shapiro-Wilk test. The normal distribution calculated yielded a mean ±2DS and 95% Confidence Interval; median and range, for skewed data (Table 2).

**Table 2:** Endometrial Cytometry Flow. Normal Reference Value

Variables - n=25	Mean ± 2SD	95% CI	*p* value
**LT/Li**	51.96±16.05	35.908 - 68.01	0.112*
**NK/Li**	40.04±14.58	25.458 - 54.62	0.157*
**CD4/Li**	44.32±7.78	36.539 - 52.1	0.324*
**CD8/Li**	56±12.31	43.6912 - 68.31	0.729*
**CD4/CD8**	0.64±0.38	0.2676 - 1.01	0.137*
**Li/Cells population**	5.6^‡^	2 - 6.27	0.000^†^
**LB/Li**	2^‡^	1 - 2.98	0.000^†^

* *p*>0.05 Normal distribution;

† *p*<0.05 Abnormal distribution (by Shapiro Wilk test); CI=Confidence interval; SD=Standard deviation;

‡ Median.

We analyzed the enFC values in the study group according to reference. The histopathological criteria for dating were those that correspond to ^Noyes et al. (1950)^. For endometrial dating in hormonal replacement therapy, the day of progesterone onset was considered as day 1. We diagnosed the CE by the presence of stromal plasma cells, with at least >5 plasma cells over 20 High Power Field (HPF), according to the nuclear chromatin rearrangement, which appeared as a clock-face or a spoke-wheel pattern, or ESPDI>0.25 ^(Johnston-MacAnanny et al., 2010; Kitaya et al., 2017; Greenwood & Moran, 1981; Eschenbach, 2008)^. We considered the following histopathological criteria: 1) superficial mucosal edematous change, 2) elevated stromal cell density, 3) fibroblast-like stromal cells (spindle stroma), 4) dissociated maturation between epithelial cells/stromal fibroblasts, 5) presence of polymorphic inflammatory cells, 6) delayed differentiation of endometrium in the mid-secretory phase, 7) pseudostratification ^(Kitaya & Yasuo, 2010; Michels, 1995; Greenwood & Moran, 1981; Eschenbach, 2008; Mishra et al., 2008; Kiviat et al., 1990)^. A normal enHP had ESPDI(0.25 in absence of above parameters or persistence of no more than three of the patterns described. The cases in which HP and IHC remission were incomplete were considered as persistent CE. The bacteriological examination consisted in fresh exam with Gram/Giemsa staining and culture on Thayer Martin medium, sheep blood agar, and chocolate agar, Saboreaud agar medium, and EMB/CLDE. We tested *Chlamydia trachomatis* using immunofluorescence; and *Mycoplasma hominis* and *Ureaplasma urealyticum* by Mycofast-urea/arginine. We defined altered contractility by the presence of the endometrial waves in midluteal phase with a frequency >4-5 contractions. 

### Endometrial therapy

We instructed all the patients to change their eating habits to a Mediterranean diet, under nutritional control as well as to refrain from toxic substances, especially nicotine, add physical activities to their routines and take vitamin supplements. We prescribed Glycine 100 mg/day associated with Vitamin E 300 mg, Vit. B6 100 mg, Vit. A 10.000 UI and Vit. D 300 IU/day. When a germ was found, we prescribed the specific antibiotic therapy for at least 30 days. When we couldn’t find a specific germ, we used oral Doxycycline (100 mg, twice per day) for 14 days, continuing with a combination of metronidazole and ciprofloxacin (both drugs in 500 mg, twice per day, 14 days) and ending with Clarithromycin (1 gr/day for 12 days). When there was no remission of the inflammatory process in the post-treatment biopsy, we repeated the scheme above and added Linezolid (600 mg/day orally for 10 days), and performed a new enHP. For mycoplasma relapses, we prescribed minocycline (100 mg, twice per day, 12 days). In patients with elevated enNK, we added methylprednisone in a dose of 2 mg/day orally. In high enNK concentrations and/or thin endometrium, we indicated intrauterine instillations of Granulocyte Colony Stimulating Factor (G-CSF) (Filgastrin), in doses of 300 micrograms at the beginning of progestin supplementation in replacement cycles. We used a second dose subcutaneously five minutes after the blastocyst transfer. Finally, in cases with increased uterine contractibility, we used an oxytocin inhibitor (Atosiban), at a dose of 6.75 mg per slow intravenous administration, prior to transfer.

### Receptor protocol

In the presence of ovarian activity, we suppressed the hypothalamus using depot GnRHa, Triptorelin 3.76 mg in a single dose, or Leuprolide acetate in doses of 200 to 300 micrograms/day, in the long regime; beginning estrogen replacement in the presence of a plasmatic estradiol <30 pg/ml. We administered Estradiol valerate and 17 β-estradiol in increasing doses orally, according to endometrial response and established protocol. Five or six days before the transfer, we added Progesterone gel in daily doses of 90 mg per vaginal via. In cases of inappropriate endometrial response, we added 17 beta estradiol transdermally, in doses of 1.5 mg/dl. We individualized all transfers according to the previous biopsy and the level of endometrial estrogen and progesterone receptors.

### Assisted Sub-hatching

We routinely performed the Tyrode technique two hours prior the transfer, according to the Hogan protocol, but not completing the solution of continuity ^(Hogan et al., 1986)^.

### Criteria for embryo transfer and intervention

Only expanded blastocysts of optimum quality (score 2.1.1, 3.1.1 or 4.1.1 with onset of hatching) were transferred, in a number no higher than two. In cases where a second blastocyst was suboptimal, the embryo transfer was allowed, with quality (2.1.2, 2.2.1, 3.1.2 or 4.2.1). Cases of single embryo transfers (SET) were considered only in optimal quality. The same criterion was applied in fresh transfers or frozen-thawed embryo transfer (FET).

### Outcome Variables

Our clinical outcome was CP and LBDR per embryo transfer cycles after the treatment, according to the definition of the included studies.

### Statistical Analysis

For the primary objective, we employed Binary Logistic Regression (BLR) to determine the independent endometrium factors that affect the prevalence of CP in RIF. A *p*-value ( 0.05 was significant and >0.05 not significant (NS). For the secondary, we assessed the clinical results in function of the normalization or no normalization of predictor in post treatment. We compared both groups using the Chi-square test and the t-test, where appropriate. We ran the statistical analyses using the SPSS version 20 (IBM Corp.,USA).

### Preparation and selection of variables for BLR.

All the variables described above represent potential predictors for CP in the post-treatment. We categorized them as normal or abnormal, CF variables in relation to the reference group, and enHP according to the mentioned criterion. We analyzed the variables to rule out confounding factors, using adjusted odds ratio, Mantel and Cochrane tests. In order to categorize the independent factors, we validated them through the Pearson Chi^2^ *p*<0.20.

### Building and validation of the BLR model

We selected the independent predictors using the statistical Wald Chi^2^. We calculated the fit adequacy using the Omnibus test. To check plausibility through estimates we applied the Nagelker R^2^ test. The degree of association between what was observed and what did the model predict, we tested using the Hosmer and Lemeshow probe. We obtained the logistic regression coefficient (Β) for the independent variables, we chose.

### General method

A prospective study of a model-based control associated with an analogical abductive methodology. The abductive method created by Peirce in 1878 ^(Houser & Kloesel, 1992)^, enlarged by ^Samaja (1999)^ and completed by ^Salatino (2009)^, who called it *adduction* ^(Salatino, 2017; Tersoglio & Salatino, 2017)^. It is the one indicated when an investigation starts with the results, as it is in this present case where our central concern is a particular element of the system focus on the endometrium.

## RESULTS

### Predictor variables

We validated the LBR model using the Omnibus test (*p*=0.00001), showing the properly adjusted data. We selected the endometrial histopathology enHP and enNK variables as predictors of Clinical Pregnancy using the Wald chi-square test with a significant *p* value (0.044 and 0.001, respectively). The odds ratio (OR) value reinforces the p we obtained (Table 3).

**Table 3:** Binary logistic regression analysis of factors affecting the Clinical Pregnancy in RIF

Parameter	β	Wald test	*p*-value	OR (IC)
Endometrial Biopsy	-1.365	4.044	0.044	0.255 (0.68-0.996)
NK/Li	-2.467	10.403	0.001	0.085 (0.19-0.380)
Regression Constant	1.766	17.312	0.000	

The model of pregnancy probability, or its accuracy degree, results in 80.03%. The Sensitivity of the model to obtain Clinical Pregnancy is 93.18% and the Specificity is 54.55%.

Finally, we calculate the predictive function with the following equation:

We built the Pregnancy Probability of Pregnancy using the ( values listed in Table 2. When both variables are normal, post-treatment results in 88.76%, and in cases of lack of standardization it is 14.60%.

### Reproductive outcome

We compared the clinical results according to the normalized or non-normalized predictor variables. There was no difference in relation to the number of embryos transferred (2.12(0.45 *vs.* 2.08(0.40; NS, respectively). The implantation rate was significantly higher in normalized vs no normalized groups (55.68 *vs.* 23.99; t-test *p*=0.0004) as well as the CP per transfer (51.5 *vs.* 15.1; Chi-square test *p*=0.0005). As expected, LBDR was significantly higher in women who normalized *vs.* their non-normalized counterparts (48.48 *vs.* 10.61, respectively, *p=*0.00009). There was no significant differences in both groups vis-a-vis the number of first-trimester miscarriages and extremely premature deliveries. There was a high percentage of prematurity in both normalized *vs.* non-normalized groups (34.4% (11/32) and 71.43% (5/7), respectively). There was a higher prematurity rate in the non-normalized *vs.* the normalized group, but the difference was not significant. (Table 4)

**Table 4:** Clinical results in relation to normalization of predictors to post-treatment

	NORMALIZED (n=41)	NO NORMALIZED (n=25)	*p*-value
Nº of embryos transferred (µ±SD)*	2.12±0.458	2.08±0.400	NS
Implantation rate (µ±SD)*	55.68±34.08	23.99±33.01	<0.001
Clinical Pregnancy (%)**	82.93 (34/41)	40.00 (10/25)	<0.001
Missed Miscarriage (%)**	4.88 (2/41)	12.00 (3/25)	NS
LBDR (%)**	78.05 (32/41)	28.00 (7/25)	<0.001
Premature Delivery (%)**	34.38 (11/32)	71.43 (5/7)	NS
Extreme Premature Delivery (%)**	54.55 (6/11)	40.00 (2/5)	NS

* t-test;

** Chi-square test

**Figure 2:**
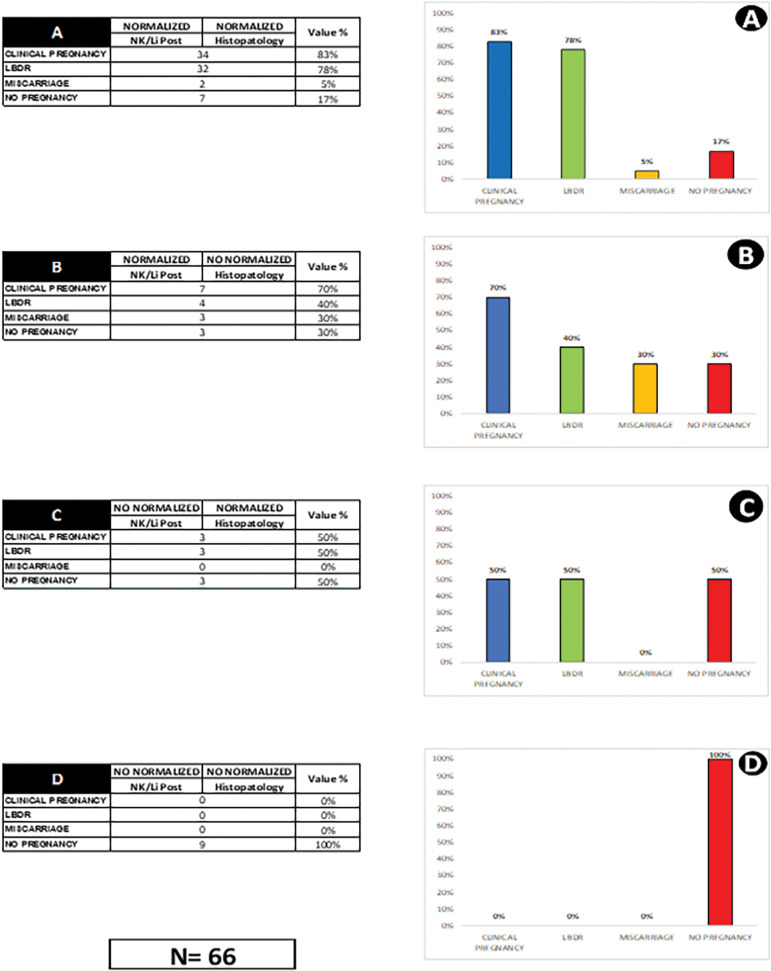
Pregnancy outcomes in post-treatment in relation to individual normalization or non-normalization of predictors

The incidence of single deliveries was 66.6% (26/39), and twin deliveries 33.3% (13/39). The incidence of twin births in normalized *vs.* non-normalized groups was not different (*p*=0.55), and half of them resulted from twin pregnancies in obstetrical management. From 52 births, half resulted from twin pregnancies. Additionally, we established the pregnancy outcome in relation to four subgroups, based on the normalization of the predictors. Group A: both predictors were normal; Group B: normal NK/Li and abnormal enHP; Group C: abnormal NK/Li and normal enHP; finally, Group D: both abnormal. We analyzed CP, LBDR and miscarriage rates individually, with the following results: Group A: 83% (34/41), 37.8% (32/41) and 5% (2/41); Group B: 70% (7/10), 40% (4/10) and 30% (3/10); Group C: 50% (3/6), 50% (3/6) and 0% (0/6), respectively; and Group D with no pregnancy (Figure 2).

## DISCUSSION

There were over 29892 egg donation cycles carried out in the world; 14647 yielded live births, which means that 51% of the cycles failed to produce a live birth ^(CDC/ASRM/SART, 2011)^. This situation continues, and it is the primary evidence of endometrium importance in IVF. The results of this study give evidence of endometrial anomalies, which may alter endometrial receptivity, thus justifying RIF. enNK and enHP normalization proved to be reliable and independent predictors of CP in an RIF population. BLR models enable a definition of the independent variables that predict clinical pregnancy. The association between CE and infertility was first reported 40 years ago, and then by multiple authors with a variable frequency of 14 to 66%, depending mainly on the diagnostic techniques applied ^(Bouet et al., 2016; Tersoglio & Salatino, 2017; Czernobilsky, 1978; Romero et al., 2004; Kamiyama et al., 2004)^. For over 106 patients with unexplained infertility and RIF, 66% were associated with CE ^(Cicinelli et al., 2015)^. In our current series, 38/66 (57.58%) presented CE and 15/66 (22.7%) had histopathological abnormal patterns, which are frequently associated with chronic endometritis. Previous studies have produced contradictory results, some of them with an increased number of enNK cells in women with recurrent miscarriages ^(Clifford et al., 1999; Quenby et al., 1999; Tuckerman et al., 2007)^ and RIF ^(Tuckerman *et al*., 2010; Laird et al., 2011)^. Others investigators suggested no difference or a reduction in the previous values, other authors do not share these criteria ^(Moffet et al., 2004; Thum et al., 2004; Bulmer & Lash, 2005; Tang et al., 2011)^. Uterine NK cells intervene in the early stages of spiral artery remodeling; and higher enNK, may induce an abnormal angiogenesis with subsequent oxidative stress ^(Quenby *et al*., 2009; Lash *et al*., 2006)^. Previous studies have shown that isolated CD56^+^ enNK cells from women with RIF produce the lowest level of angiogenic factors, such as vascular endothelial growth factor (VEGF), placental growth factor (PLGF) and platelet-derived growth factor (PDGF-BB), compared with women with RM and fertile controls ^(Chen et al., 2016)^.

On the other hand, an increased cytotoxic subpopulation of CD56- CD16+ in a population with recurrent abortions may be an expression of chronic or latent infection ^(McQueen et al., 2015)^. While it is true that stromal PCs is the most substantial landmark in the diagnosis of CE ^(Kitaya & Yasuo, 2011; Akopians et al., 2015)^, associated immunohistochemical staining methods enable higher diagnostic accuracy ^(Kitaya & Yasuo, 2013; El-Toukhy et al., 2008; Bayer-Garner et al., 2004)^, although one should not consider it an axiom. The delayed differentiation of endometrium in the mid-secretory phase is one of the histopathologic characteristics seen in infertile patients with CE. The glandular epithelium and surface epithelial cells in CE often display pseudo-stratification and unusual mitotic activity -a probable explanation of the high level of estrogen receptor in CE ^(Mishra et al., 2008)^. The latter could lead us to detect the presence of compatible cases or at least suspected cases of CE, which is usually associated with a large number of focal LB infiltrate in the stromal endometrium. Selectin E plays a crucial role in attracting B cells by extravasation and migration into endometrial epithelium and glandular lumina. On these pathophysiological bases, an interesting model of pathogenesis has been proposed in CE, which may explain the local differentiation into PCs. As a result, a decrease of endometrial receptivity associated with genes, resulted in delayed endometrial differentiation ^(Kitaya *et al*., 2016)^.

Earlier studies focused on the potential role of *Chlamydia trachomatis* and *Neisseria gonorrhea* as the main pathogenic microorganisms causing CE. However, a randomized clinical trial demonstrated a low detection rate of *C. trachomatis* (7%) and *N. gonorrhea* (8%) in women with CE ^(Haggerty et al., 2004)^. Recent studies describe the preponderance of Gram(-) and Mycoplasma/Ureaplasma in endometrial culture from an RIF population ^(Cicinelli et al., 2015)^. Our cases show a positive microorganism identification in 19/66 (28.8%), with an incidence of *Mycoplasma genitalium* and *Ureaplasma urealyticum* of 8/19 (42%), *Chlamydia* 6/19 (31.6%) and G(-)&G(+) 5/19 (26.6%). It is likely that the poor bacteriological identification rate could be associated with the use of a conventional bacteriological method ^(Cicinelli *et al*., 2012)^. The interaction between microbes with endometrial epithelium could alter the mucosal immunity by several mechanisms. The latter may include altered endometrial gene expression encoding proteins involved in the inflammatory response, proliferation/apoptosis, abnormal expression of leukocyte subsets, abnormal infiltration of plasma cells and secretion of immunoglobulin. Therefore, these events may finally impair different processes as abnormal decidualisation or give rise to pathological conditions, like the CE ^(Kitaya et al., 2014)^. An example of the above is the case of *Chlamydia trachomatis* that produces a protein (plasmid-encoded Pgp3) that neutralizes the induced anti-chlamydial activity of the LL-37 peptide ^(Hou et al., 2019)^. Recently, studies described the importance of Lactobacillus dominance in the uterine microbiota as a factor associated with IVF success ^(Moreno et al., 2016; Moreno & Franasiak, 2017)^. Although it is well accepted that endometrial microbiome analysis represent one of the most exciting and interesting acquisitions ^(D’Ippolito et al., 2018)^, culture-based microbial studies show several limitations, since <1% of bacteria reliably grow and form colonies, even in appropriate culture conditions, they do not represent the “diversity” of a particular microbiome ^(Verstraelen et al., 2016)^. These reasons are sufficient to validate the use of antibiotics, even in the absence of microbial identification, requiring in many cases prolonged application until the standardization of endometrial variables.

There was a high association of RIF with thinned endometrium of 23/66 (34.85%), of which only 7/23 (30.43%) recovered after treatment. In the presence of persistent endometrial thickness associated or not with an increase in enNK, it was associated with the use of G-CSF in the transfer cycle. In a recent meta-analysis, G-CSF perfusion improve endometrial thickness, clinical pregnancy and embryo implantation ^(Xie et al., 2017)^. G-CSF promotes the increase in regulatory T-cells ^(Rutella et al., 2005)^, activating dendritic cells ^(Adusumilli et al., 2012)^, suppressing the cytotoxic activity of NK cells ^(Taga et al., 1993; Schlahsa et al., 2011)^, mediating the shift of Th1/Th2 ratio in favor of a Th2 response ^(Sloand et al., 2000)^, even though the mechanism of G-CSF on endometrial thickness remains obscure and needs further investigation. Post-treatment normalization of NK/Li and enHP were independent predictors of CP in this study. The BLR model enabled us to determine, with a high degree of sensitivity and moderate specificity, to identify independent variables that predict clinical pregnancy. The persistence of CE requires a special consideration; 19/66 (28.78%) did not achieve enHP normalization. The prevalence of CE persistence showed similar levels to those reported by ^Cicinelli et al. (2015; 2018)^ (24.6 and 17.6%, respectively), but different from those reported by ^Kitaya et al. (2017)^ (0.85%). The inconsistency among the studies is probably due to the various diagnostic tools used in CE and RIF studies (microbiology, associated with histopathology, hysteroscopy, CF and IHQ).

The posttreatment normalized predictors resulted in significantly higher implantation rates, clinical pregnancy, and LBR compared with non-normalized groups. The normalization of both predictors results in higher CP and LBDR, and a lower abortion rate (Fig. 2A). In contrast, the absence of normalization of both predictors results in absence of pregnancy (Fig. 2D). Apparently, in this series, the incidence of miscarriage is associated with enHP, the highest miscarriage was found in abnormal enHP (Fig. 2B). Previous studies suggest a relationship between pre-existing inflammatory local conditions and premature birth ^(Ghidini & Salafia, 2005)^. Concerning our RIF population, there was a high frequency of premature deliveries, 16/39 (41%) of which presented extreme prematurity. Interestingly, there was no significant difference between normalized/non-normalized in premature deliveries. These findings suggest some questions about the cure criteria or if necessary, to adopt other strategies in obstetrical manage. On the other hand, a higher frequency of premature births in oocyte donation has been published, suggesting a different modulated immune activity at the maternal-fetal interface of egg-donor pregnancies ^(Gundogan et al., 2010)^. Probably the advent of the endometrium regenerative cell therapy appears to be an efficient tool in endometrial immunology therapy ^(Tersoglio et al., 2020)^. The strength of this research is based on the strict follow-up in enHP, enIHQ and the immunological shifts before/after treatment. The construction of the normal reference values in enFC and the predictive capacity, confirmed by the clinical results. The clinical implications of RIF associated with untreated CE not only refer to the poor results in IVF, but also delays the treatment resolution. In our view, the normalization of an affected endometrium requires negative endometrial bacteriology, a normal cytometric profile and normal endometrial biopsy.

Our studies show that the endometrial standardization requires unfixed periods with a length therapy (from 42 to 168 days). The probability of pregnancy was estimated in 80%, and in the presence of normalized variables de CP was 88.76%. Future investigations should clarify the following questions: What other aspects should be considered to define a cured CE? Which of the bacteriological, immunological or histopathological criteria should be applied? How to manage the obstetrical follow-up to reduce adverse perinatal outcomes?

## CONCLUSION

- The enHP and the enNK/Li ratio showed that they are valid predictors of CP in RIF. The model in 80% estimated the probability of pregnancy, and in presence of both normalized predictors, it results in 88.76%.
- The outcome pregnancy (IR, CP and LBDR) result was significantly higher in normalized predictors vs. non-normalized in post-treatment. There was no significant difference in relation to miscarriage. There was a higher prematurity in the non-normalized group, but the difference was not significant.
- The endometrial therapy and the normalization of predictors were unable to achieve a significant decrease in the prematurity rate.
- The pregnancy outcomes in post-treatment related to individual predictors show:
  - If both predictors were normalized, there was a high CP with low abortion.
  - normalized enNK and non-normalized HP present a high CP and miscarriage.
  - Non-normalized enNK and normalized HP present an acceptable CP and LBDR without miscarriage.
  - If both predictors were not normalized, there was absence of pregnancy.

The reversibility of endometrial histological changes, as endometrial deleterious immunological aspects, could be a strategy to achieve a live birth in RIF under polyvalent therapy. Finally, the merit of this study was to provide a clinical tool to optimize the chances of pregnancy in an RIF population with high proportion of CE.
